# Supplementary material for: Identification of photoperiod-induced specific miRNAs in the adrenal glands of Sunite sheep (Ovis aries)
Source: Front Vet Sci. 2022 Jul 22;9:888207. doi: 10.3389/fvets.2022.888207 (PMC9354845; doi:10.3389/fvets.2022.888207)
Supplement: Supplementary file 1 [file Data_Sheet_1.ZIP › Supplementary file/miRDeep2 file.zip!/miRDeep2 file/Know_Structure/LP42A2/oar-mir-433.pdf]

miRBase precursor : oar-mir-433
Total read count : 905
oar-miR-433-5p read count : 2
oar-miR-433-3p read count : 903
remaining reads : 0

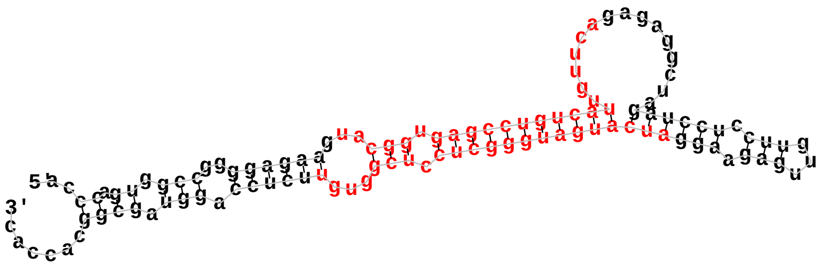

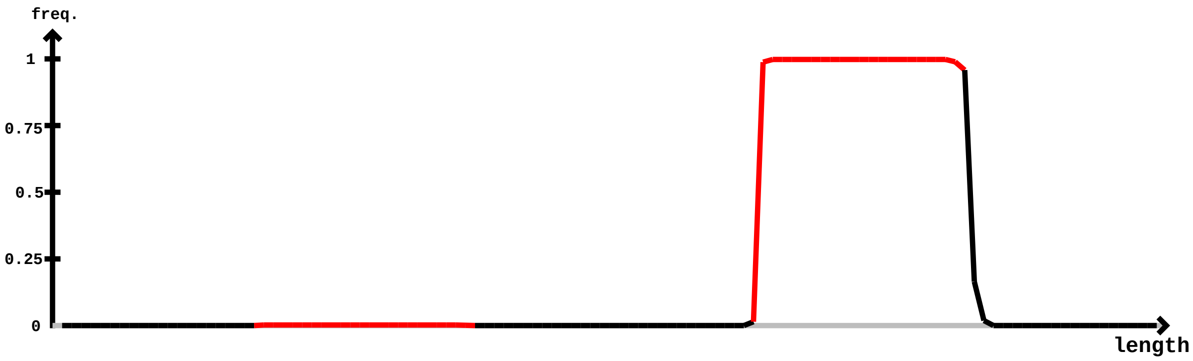

oar-miR-433-5p

oar-miR-433-3p

```
5'- acccaguggccggggagaaguacggugagccugucauuguucagagaggcuagauccuccuuguugagaaggaucaugaugggcuccucgguguucuccagguagcggcaccac   -3'    exp
    ..((.((.(((..((((((...(((.(((((((((((...............((((((.(((...))).))))))))))))))))).))).)...)))))).))).))))......    reads    mm    sample
    ....................uacggugagccugucauuguu.........................................................................    1        0     seq
    ....................uacggugagccugucauuguuc........................................................................    1        0     seq
    .......................................................................gaucaugaugggcuccucggugu....................    13       0     seq
    ........................................................................aucaugaugggcuccucggu......................    8        0     seq
    ........................................................................aucaugaugggcuccucggug.....................    28       0     seq
    ........................................................................aucaugaugggcuccucggugu....................    697      0     seq
    ........................................................................aucaugaugggcuccucgguguu...................    132      0     seq
    ........................................................................aucaugaugggcuccucgguguuc..................    16       0     seq
    .........................................................................ucaugaugggcuccucggugu....................    8        0     seq
    .........................................................................ucaugaugggcuccucgguguuc..................    1        0     seq
```
